# Perindopril-induced angioedema of the lips and tongue: a case report

**DOI:** 10.1186/s13256-018-1910-x

**Published:** 2018-12-05

**Authors:** Fawaz Abdullah Alharbi, Abdulwahab Awadh Alharthi, Faisal Nuefa Alsaadi

**Affiliations:** 1 Drug Information and Poison Center, Alansar Hospital, Al Madinah, Al Munawarah, Saudi Arabia; 2 Internal Medicine Department, Alansar Hospital, Al Madinah, Al Munawarah, Saudi Arabia; 3 Pharmaceutical Care Department, Alansar Hospital, Al Madinah, Al Munawarah, Saudi Arabia

**Keywords:** Angioedema, ACE inhibitor-induced, Lips, Tongue, Angiotensin-converting enzyme inhibitor, Perindopril

## Abstract

**Background:**

Ace inhibitor-induced angioedema, characterized by sudden-onset swelling of the mucous membrane, skin, or both, is a rare occurrence in the Kingdom of Saudi Arabia. Because of its safety and efficacy, perindopril is a commonly prescribed angiotensin-converting enzyme inhibitor. Here we describe the clinical manifestations, management, and outcome of perindopril-induced angioedema of the lips and tongue in a 65-year-old Saudi man.

**Case presentation:**

A 65-year-old Saudi Arab man presented to an emergency department with lip and tongue swelling and dysphagia. There were no systemic symptoms and no past history of a similar event. He had been consuming perindopril 5 mg and amlodipine 5 mg for the last 3 weeks: brand name, Coveram, from the company Servier (Ireland) Industries Ltd.; one tablet of Coveram contains 3.395 mg perindopril corresponding to 5 mg perindopril arginine and 6.935 mg amlodipine besilate corresponding to 5 mg amlodipine. A physical examination revealed considerable swelling of his lips and tongue.

Examinations of other systems, including his cardiovascular and respiratory systems, revealed unremarkable findings. All laboratory parameters were also normal. Electrocardiography demonstrated sinus rhythm, a normal P axis, and V-rate of 50–99.

A clinical diagnosis of perindopril-induced angioedema was made, and perindopril was discontinued. The angioedema resolved completely after the administration of antihistamines and corticosteroids.

**Conclusions:**

Angioedema caused by angiotensin-converting enzyme inhibitors is an uncommon occurrence in Saudi Arabia. The findings from this case are expected to raise awareness about this condition among clinicians in this country.

## Background

Although angioedema was first explained centuries ago, the etiology, pathophysiology, and treatment of several forms of this condition remain unclear. Hereditary angioedema is basically a genetic problem caused by the malfunction or deficiency of C1 esterase inhibitor. It is an autosomal dominant condition characterized by non-pitting, non-pruritic, and painless swelling of the skin [1]. On the other hand, acquired or nonhereditary angioedema is the consequence of acquired insufficiency of C1 esterase inhibitor, unnecessary stimulation of the contact–kinin system, and overactivation of the human complement system [2]. It can also be idiopathic or the result of an allergic reaction to drugs, food, autoimmune disorders, or several inhalants [3]. Nonhereditary angioedema recurs at random intervals, persists for 2 days or more, and involves different parts of the body, including the face, abdomen, genitals, and limbs. It can be life-threatening if it involves the oral mucosa and upper respiratory tract.

Angiotensin-converting enzyme (ACE) inhibitors are considered one of the most frequent causes of acquired angioedema, accounting for 25–40% of all cases of angioedema [3]. However, other drugs such as nonsteroidal anti-inflammatory drugs (NSAIDs), aspirin, certain antibiotics, and angiotensin II receptor antagonists can also result in this condition. ACE inhibitor-induced angioedema primarily occurs because of the enzymatic inhibition of bradykinin degradation [4]. The incidence of angioedema caused by the use of ACE inhibitors has been increasing over the last two decades [5]; this is because these drugs are consumed by individuals worldwide for the management of heart failure, renal failure, myocardial infarction, and nephropathy due to diabetes mellitus [5].

Here we describe the clinical manifestations, management, and outcome of perindopril-induced angioedema of the lips and tongue in a 65-year-old Saudi man. This case is unusual because angioedema caused by perindopril, a known ACE inhibitor, is an unusual occurrence in Saudi Arabia.

So far, only four cases of angioedema caused by ACE inhibitors have been reported to the National Pharmacovigilance and Drug Safety Center at the Saudi Food and Drug Authority by different hospitals in Saudi Arabia. In 2015, Bukhari *et al.* [6] reported the first study regarding the occurrence of angioedema following administration of ACE inhibitors in a 9-year-old girl with renal disease. The rarity of this condition in Saudi Arabia may be because the predisposing factors are still unknown, although it is more common among African-American patients.

## Case presentation

A 65-year-old Saudi Arab man presented to an emergency department with lip and tongue swelling and dysphagia. There were no other systemic symptoms. He was taking perindopril and reported no history of problems after the intake of ACE inhibitors for hypertension. There was no other associated background, no drug-related or food-related allergies, and no previous history of similar episodes. His medical history included essential hypertension and benign prostatic hyperplasia, and he had consumed one tablet of perindopril 5 mg and one tablet of amlodipine 5 mg daily over the previous 3 weeks, along with one tablet of prolonged-release alfuzosin 10 mg daily over the previous 6 months. He had never taken any over-the-counter medications or herbal supplements, and his family history was insignificant for similar allergies or atopy.

There was no complaint of headache, fainting, dizziness, shortness of breath, chest pain, or any cardiac problem. On examination, cyanosis was absent. His bowel and urinary habits were normal, and there was no known clotting or blood disorder and no endocrine abnormality. He was financially stable; he was a non-tobacco smoker; he was generally alert and active.

A physical examination revealed considerable swelling of his lips and tongue. He was well oriented in time, place, and person. His vital signs were as follows: temperature, 37 °C (98.6 °F); heart rate, 101 beats/minute; respiratory rate, 22 breaths/minute; oxygen saturation, 99%; and blood pressure, 147/88 mmHg.

There was no abnormal pulse or palpable lymph node, and examinations of other systems revealed unremarkable findings. His skin was normal in appearance, temperature, and texture, and there was no rash or pruritus. There was no dull sound on percussion, and normal ventral breathing was observed on auscultation. Inspiratory stridor, wheeze, and rhonchi were absent. An examination of his cardiovascular system revealed normal heart sounds SI and S2 (S1 + S2 + 0), without any thrills or heaves. His abdomen was soft, non-tender, and symmetrical, while his bowel sounds were normal in intensity and quality in all areas. No splenomegaly or masses were noted; his liver span was found to be 9 cm on percussion.

All laboratory values were within normal limits. Electrocardiography demonstrated sinus rhythm, a normal P axis, and V-rate 50–99. However, abnormal R-wave progression and early transition were observed, with the QRS area > 0 in v2.

A clinical diagnosis of perindopril-induced angioedema was made, and perindopril was discontinued. In the emergency department, intravenously administered hydrocortisone 200 mg and 1 L of normal saline was administered along with intravenously administered ranitidine 50 mg and intramuscularly administered magnesium 25 mg and promethazine 50 mg. Subsequently, he was nebulized with salbutamol 5 mg and admitted to a medical ward, where he was fully conscious and oriented, with the following vital signs: temperature, 37 °C (98.6 °F); heart rate, 104 beats/minute; respiratory rate, 22 breaths/minute; oxygen saturation, 99%; and blood pressure, 147/88 mmHg. Chest auscultation revealed normal findings, with normal ventral breathing without wheezing.

He received the following medications in the ward: amlodipine 5 mg orally administered once daily, chlorpheniramine maleate 10 mg orally administered once daily, ranitidine 150 mg orally administered twice daily, hydrocortisone 100 mg intravenously administered three times daily, promethazine 50 mg intramuscularly administered twice daily, and paracetamol 1 g intravenously administered as needed.

His lip and tongue swelling gradually subsided, and he remained in the hospital for only 1 day. He was discharged when his symptoms alleviated and he felt better. He was advised against the consumption of any type of ACE inhibitor. At the time of discharge, the following medications were prescribed: amlodipine 5 mg orally administered once daily, ranitidine 150 mg orally administered twice daily, and prednisolone 10 mg orally administered twice daily for 2 days. He was recalled after 7 days. At the follow-up visit, he did not complain of any recurrence.

The probability of perindopril causing an adverse event in a patient was assessed using the Naranjo Adverse Drug Reaction Probability Scale, which systematically eradicated all other possible etiologies for a reaction and correlated with the onset of symptoms associated with suspected drug use [7]. His score indicated a probable association between perindopril and angioedema of the lips and tongue.

## Discussion and conclusions

We reported a case of perindopril-induced angioedema of the lips and tongue in a 65-year-old Saudi man. Angioedema is a potentially life-threatening condition characterized by well-demarcated, sudden-onset swelling of the deeper layers of the skin and mucous membranes [8]. The same clinical manifestation was observed in our patient. However, according to Kanani *et al.*, angioedema may commonly involve other sites like the lips, face, tongue, hands, eyes, feet, bowel, and genitalia [9]. Edema of the larynx, pharynx, and tongue also occurs, which is particularly challenging to treat. This edema is normally skin-colored, non-pitting, and non-itchy; however, it can occur along with urticaria [9]. In the present case, edema remained confined to the lips and tongue and was non-pitting and non-itchy.

Angioedema is the result of a quick increase in the permeability of capillaries and venules, with localized extravasation of plasma. The swelling normally lasts from a few hours to 72 hours. Kulthanan *et al.* found a number of etiologies responsible for inducing the release of vasoactive substances, such as bradykinin or histamine [10], which are also involved in allergic reactions to various foods, drugs, or insect bites. In this particular case, the major cause was the use of ACE inhibitors. In addition to ACE inhibitors, angioedema is classified into several types, including allergic, idiopathic, NSAID-induced, hereditary, and ACE inhibitor-induced [11]. However, among all of these causes, that angioedema can be induced by ACE inhibitors is well recognized [12].

ACE inhibitors are broadly used for the management of hypertension, myocardial infarction, heart failure, diabetic nephropathy, and renal failure, and their use has considerably increased over the last couple of decades. At present, approximately 40 million individuals worldwide are receiving treatment with ACE inhibitors; this could result in a greater incidence of angioedema [13]. Knowledge about the association between drug intake and the occurrence of angioedema is important for timely recognition of the condition and subsequent discontinuation of the causative drug.

The prevalence of ACE inhibitor-induced angioedema globally is 0.1 to 0.7%, and it is more common among individuals of African descent [14]. Although it occurs episodically, every event follows a comparatively predictable interval of 2 to 5 days. Swelling generally develops within a few minutes or several hours; the swelling subsides within 1 to 3 days. In some cases, complete resolution may take some time even after discontinuation of the causative drug. Angioedema due to ACE inhibitors can involve not only the lips, face, and tongue but also the subglottic region, larynx, pharynx, and intestine [15, 16]. In patients with renal impairment, the intensity of edema may be severe because of considerable drug build-up.

The precise mechanism by which ACE inhibitors induce angioedema remains unknown, but bradykinin is considered to play a role [17]. ACE inhibitors inhibit the actions of ACE, also identified as kininase II, thus affecting the renin–angiotensin–aldosterone system as well as the breakdown of bradykinin. Bradykinin can subsequently lead to vasodilatation and capillary leakage, resulting in angioedema.

In this case, ACE inhibitor-induced angioedema was diagnosed clinically because of the unavailability of specific laboratory tests. In general, this type of angioedema is self-resolving and responds well to discontinuation of the causative drug, which also confirms the diagnosis. In the present case, our patient was a chronic user of ACE inhibitors and had no comorbidity. On the other hand, patients with severe edema, particularly in the upper airway, may require either mechanical ventilation or an invasive procedure such as tracheotomy, intubation, or cricothyroidotomy [18, 19]. However, in this patient the administration of antihistamines and corticosteroids brought about a resolution.

This problem is an atypical occurrence in Saudi Arabia, as only one case has been reported [6]. Moreover, when we inquired of the National Pharmacovigilance and Drug Safety Center at the Saudi Food and Drug Authority regarding this, they stated that only four cases of angioedema induced by either lisinopril, captopril, or perindopril have been documented. Therefore, we believe that this case report will provide valuable information to clinicians in Saudi Arabia.

In conclusion, ACE inhibitor-induced angioedema is a potentially lethal condition, and early detection is necessary for appropriate therapeutic intervention. Emergency physicians, particularly those working in health care settings in Saudi Arabia, must be aware about this form of angioedema. Early diagnosis cannot only resolve a patient’s symptoms, but also prevent the condition from becoming life-threatening. Precise treatment involves the discontinuation of the causative ACE inhibitor and close monitoring. Further studies are necessary, principally in countries like Saudi Arabia, for more awareness and an increased understanding regarding this condition.

## Abbreviations

ACE: Angiotensin-converting enzyme inhibitor
NSAIDs: Nonsteroidal anti-inflammatory drugs
